# Mental Health Impacts of Multidrug-Resistant Tuberculosis in Patients and Household Contacts: A Mixed Methods Study

**DOI:** 10.7759/cureus.60412

**Published:** 2024-05-16

**Authors:** Yogesh Murugan, Nirmalkumar Patel, Vinay Kumar, Rohankumar Gandhi

**Affiliations:** 1 Family Medicine, Guru Gobind Singh Government Hospital, Jamnagar, IND; 2 Preventive Medicine, Guru Gobind Singh Government Hospital, Jamnagar, IND; 3 Preventive Medicine, Shri M. P. Shah Medical College, Jamnagar, IND; 4 Community and Family Medicine, Shri M. P. Shah Medical College, Jamnagar, IND

**Keywords:** stigma, household contacts, anxiety and depression, depression, multidrug-resistant tuberculosis

## Abstract

Background: Multidrug-resistant tuberculosis (MDR-TB) patients experience disproportionately worse mental health, with implications for adherence, outcomes, and families. Comprehensive assessments of comorbid depression/anxiety and related factors remain limited.

Objective: This study aimed to assess the prevalence, predictors, and qualitative experiences of depression and anxiety in MDR-TB patients and household contacts.

Methods: A sequential explanatory mixed methods study was conducted in Gujarat, India, with 403 smear-positive MDR-TB patients and 403 contacts. The quantitative phase administered structured questionnaires on sociodemographic factors, clinical history, depression/anxiety symptoms, and psychosocial stressors (like stigma and social support). Logistic regression models were used. The qualitative phase included in-depth interviews with 30 purposively sampled patients for thematic content analysis. Results were integrated to contextualize quantitative findings.

Results: High rates of depression (37.5%, n = 151) and anxiety (45.2%, n = 182) were documented among the MDR-TB patients, significantly greater than household contacts (20.1%, n = 81 and 25.1%, n = 101, respectively). For depression, older age (adjusted odds ratio (AOR) 2.03, 95% CI 1.01-4.05), female gender (AOR 2.5, 95% CI 1.1-6.0), divorced/widowed status (AOR 3.8, 95% CI 1.1-8.0), financial constraints, substance abuse (AOR 1.7, 95% CI 1.1-2.7), greater disease severity (AOR 1.8, 95% CI 1.5-2.2), medication side effects (AOR 2.4, 95% CI 1.2-4.6), and perceived stigma (AOR 3.2, 95% CI 1.1-5.3) emerged as significant risk factors. For anxiety, significant predictors were less social support (AOR 0.81, 95% CI 0.71-0.86), higher perceived stigma (AOR 2.2, 95% CI 1.1-6.3), greater disease severity (AOR 2.6, 95% CI 1.3-4.0), and more medication side effects (AOR 3.3, 95% CI 1.1-5.5). Prominent themes included psychological impacts like depression and anxiety, experiences of stigma and caretaking challenges, and recommendations for comprehensive patient support services.

Conclusion: MDR-TB patients experience a substantially higher dual disease burden of depression and anxiety, elevating the risk for adverse outcomes and transmission. Improving psychosocial support is vital to patient-centric care pathways for vulnerable groups. Mixed methods provide comprehensive evidence to inform integrated physical and mental health services.

## Introduction

Multidrug-resistant tuberculosis (MDR-TB), defined as tuberculosis caused by bacteria resistant to isoniazid and rifampicin, poses a major global health challenge [1]. Recent evidence suggests that individuals with MDR-TB face a high risk of developing comorbid mental health disorders, such as depression and anxiety [2].

Household contacts of MDR-TB patients also face a substantial mental health burden, given the psychosocial and caregiving challenges posed by the disease for families. In addition, a study in China showed that the prevalence of anxiety and depression in TB patients is about 18.37% and 18.13%, respectively [3]. Evidence indicates that TB household contacts are a high-risk group warranting greater focus, given the potential for secondary infections and transmission [4].

The COVID-19 pandemic has further amplified challenges in the provision of uninterrupted care for drug-resistant TB patients in most resource-constrained settings, such as India [5]. Emerging evidence suggests that preexisting mental health conditions can worsen due to factors, such as fear of exposure, isolation, loss of livelihood, and heightened stigma [6]. This underscores the urgency of integrated medical and psychosocial support services tailored to the unique needs of vulnerable MDR-TB patients.

Establishing the most salient drivers is essential for informing targeted mental health interventions for at-risk MDR-TB patients and contacts. Our study aimed to address this gap by analyzing and comparing the prevalence, sociodemographic correlates, and independent predictors of depression and anxiety disorders among MDR-TB patients and their household contacts and qualitative experiences of depression and anxiety in MDR-TB patients in Gujarat. These findings will inform targeted recommendations on patient-centric interventions and health policies for ameliorating disease burdens.

This article was previously posted to the medRxiv preprint server on February 12, 2024.

## Materials and methods

Study design and participants

This exploratory sequential mixed methods study involved collecting and analyzing both quantitative survey data and qualitative interview data in two consecutive phases. The purpose was to use the qualitative findings to expand on and clarify the initial quantitative results from 403 MDR-TB patients and 403 household contacts in Gujarat, India.

Sample size calculation

The sample size was determined using Epi Info 7 StatCalc (CDC; Atlanta, Georgia) for population surveys based on an estimated incidence of depression of 37.5% among MDR-TB patients from previous studies [7], a 5% margin of error, a 95% confidence level, and a 10% nonresponse rate. This generated a minimum sample size of 369 MDR-TB patients. With a 1:1 ratio of patients to household contacts, the total sample size was 738 participants. We recruited 403 participants in each group (n = 806) to account for possible missing data. For the qualitative component, we included 30 participants (15 MDR-TB patients and 15 HHCs).

Sampling technique

A consecutive sampling technique was used for patient recruitment. All eligible and consenting MDR-TB patients attending the clinics during the study period were recruited until the required sample size was reached. For each enrolled patient, one household contact was then recruited using simple random sampling from their household roster.

The study enrolled participants who met specific inclusion criteria, including being 18 years of age or older, being diagnosed with MDR-TB through drug sensitivity testing, residing with at least one household contact, and providing informed consent. Conversely, individuals were excluded from the study if they were unable to complete interviews or were diagnosed with a psychiatric disorder requiring treatment.

Data collection tools

Data collection was facilitated through meticulously designed tools, such as a sociodemographic questionnaire capturing essential details, such as age, sex, marital status, education, occupation, and income. In addition, a clinical questionnaire was used to collect comprehensive information on medical history, TB history, treatment status, and potential side effects.

To assess psychological well-being, validated scales with good internal consistency were employed: The Patient Health Questionnaire-9 (PHQ-9) was used for depression screening [8]. It is a nine-item scale with scores ranging from 0 to 27, with higher scores indicating greater depression severity. The PHQ-9 has demonstrated high internal reliability (Cronbach's α = 0.89).

The Hamilton Anxiety Rating Scale (HAM-A) was used for anxiety assessment [9]. This clinician-rated scale consists of 14 items, with scores ranging from 0 (no anxiety) to 56 (severe anxiety). The HAM-A has shown good internal consistency (Cronbach's α = 0.92).

Social support was evaluated using the Multidimensional Scale of Perceived Social Support (MSPSS) [10], a 12-item scale assessing perceived support from family, friends, and significant others, with higher scores reflecting greater support. It has high internal reliability (Cronbach's α = 0.88).

The Chronic Illness Anticipated Stigma Scale (CIASS) gauged perceived stigma [11]. This 12-item scale measures expectations of discrimination due to chronic illness, demonstrating good internal consistency (Cronbach's α = 0.93).

These robust data collection instruments with established psychometric properties enabled a thorough exploration of the participants' experiences and provided a solid foundation for the investigation. This was followed by qualitative in-depth interviews of a subset of 30 participants exploring personal experiences until saturation.

Data collection procedures

Patients were recruited at clinic visits after providing written informed consent. Trained research assistants administered questionnaires in face-to-face interviews lasting approximately 30-45 minutes. The PHQ-9 and HAM-A scores were categorized based on validated cut-offs to determine the prevalence of depression and anxiety symptoms [8,9].

Qualitative data were coded for key themes related to disease impacts, coping challenges, stigma experiences, and patient recommendations through thematic content analysis. Results were then converged by comparing and contrasting findings to achieve complementarity.

Data analysis

The data were analyzed using IBM SPSS Statistics for Windows, Version 25.0 (released 2017, IBM Corp., Armonk, NY). Descriptive statistics summarized sociodemographic and clinical characteristics. The prevalence of depression and anxiety was reported. Bivariate analysis assessed outcome-related factors using t-tests/ANOVAs and chi-square tests. Variables with p < 0.25 were entered into multivariable logistic regression models to identify independent predictors of depression and anxiety. Qualitative data was analyzed using thematic analysis (using NVivo (QSR International, USA)).

Ethical considerations

Ethical approval was obtained from the Institutional Review Board of Shri M. P. Shah Medical College and Guru Gobind Singh Government Hospital (approval ref. no. 221/04/2023) before the data were collected. Written informed consent was obtained from all participants. Confidentiality was maintained by deidentifying the data. Participation was voluntary, and the participants had the right to withdraw at any time. Referrals were provided for those requiring mental health services.

## Results

Table 1 outlines the key sociodemographic and health characteristics of the 806 study participants, including 403 MDR-TB patients and 403 household contacts. The mean age was 43.5 ± 11.4 years, 56.3% (N = 453) of the patients were males, and 65% (N = 520) were married. For the MDR-TB patients, the mean disease severity score was 7.2 ± 2.1, 36.2% (N = 292) had a low income, and 27% (N = 219) had chronic conditions.

**Table 1:** Sociodemographic characteristics of the participants, MDR-TB: multidrug-resistant tuberculosis, SD: standard deviation, BG: Bradshaw groups (classification of socioeconomic status in India), NA: not applicable

Characteristic	MDR-TB patients (n = 403)	Household contacts (n = 403)	Total (N = 806)
Age (years), mean ± SD	45.2 ± 10.3	41.7 ± 12.1	43.5 ± 11.4
Gender, n (%)			
Male	252 (63%)	201 (50%)	453 (56.2%)
Female	151 (37%)	202 (50%)	353 (43.8%)
Marital status, n (%)			
Married	249 (62%)	271 (67%)	520 (65%)
Single	102 (25%)	81 (20%)	183 (23%)
Divorced/widowed	52 (13%)	51 (13%)	103 (12%)
Socioeconomic status, n (%). By modified BG Prasad's classification:			
Low (class 3-5)	151 (37.5%)	141 (35%)	292 (36.2%)
High (class 1, 2)	252 (62.5%)	262 (65%)	514 (63.8%)
Social support score, mean ± SD	15.2 ± 5.1	16.8 ± 4.7	16.0 ± 5.0
Perceived stigma score, mean ± SD	18.5 ± 6.2	14.2 ± 5.8	16.4 ± 6.2
Substance abuse, n (%)	101 (25%)	88 (21%)	189 (23.4%)
Disease severity score, mean ± SD	7.2 ± 2.1	NA	NA
No. medication side effects, mean ± SD	3.1 ± 1.8	NA	NA
Chronic conditions, n (%)	121 (30%)	98 (24%)	219 (27%)

Table 2 shows the prevalence of depression and anxiety. The prevalence of depression based on a PHQ-9 score ≥10 was 37.5% among the MDR-TB patients and 20% among the contacts. The prevalence of anxiety per HAM-A score ≥10 was 45% in the MDR-TB patients versus 25% in the household contacts, indicating a significantly greater mental health burden in the patient group.

**Table 2:** Prevalence of depression and anxiety among the MDR-TB patients and their contacts MDR-TB: multidrug-resistant tuberculosis, PHQ-9: Patient Health Questionnaire-9 (screening tool for depression), HAM-A: Hamilton Anxiety Rating Scale

Condition	MDR-TB patients (n = 403)	Household contacts (n = 403)	Total (N = 806)
Depression (PHQ-9 ≥10), n (%)	151 (37.5%)	81 (20.1%)	232 (28.8%)
Anxiety (HAM-A ≥10), n (%)	182 (45.2%)	101 (25.1%)	283 (35.1%)

Table 3 shows the bivariate analysis of factors associated with depression and anxiety. Multiple participant characteristics, including older age, female sex, divorced/widowed status, low income, less social support, greater perceived stigma, preexisting illness history, substance abuse, and greater disease severity or medication side effects, demonstrated significant univariate associations with mental health outcomes (p < 0.05).

**Table 3:** Bivariate analysis of factors associated with depression and anxiety * only for MDR-TB patients, p < 0.05 = significant. N: number, p-value: probability value (statistical significance). The * symbol likely indicates that those factors (disease severity, medication side effects) were only measured/applicable for the MDR-TB patient group, not the household contacts.

Factor	Depression (N = 232)	No depression (N = 574)	p-value	Anxiety (N = 283)	No anxiety (N = 523)	p-value
Age	43.7 ± 9.8	38.9 ± 10.1	0.001	41.3 ± 8.7	39.2 ± 9.3	0.02
Gender						
Male	151 (33.3%)	302 (66.7%)	0.03	162 (35.5%)	291 (64.5%)	0.01
Female	81 (23%)	272 (77%)		121 (34.3%)	232 (65.7%)	
Marital status						
Married	139 (26.9%)	381 (73.1%)	0.04	178 (34.6%)	342 (65.4%)	0.06
Single	48 (26.2%)	135 (73.8%)		58 (31.7%)	125 (68.3%)	
Divorced/widowed	45 (44%)	58 (56%)		47 (45.6%)	56 (54.4%)	
Income level						
Low	168 (57.5%)	124 (42.5%)	0.01	182 (62%)	110 (38%)	0.03
High	64 (12.4%)	450 (87.6%)		101 (20%)	413 (80%)	
Perceived social support	16.4 ± 4.8	19.6 ± 4.9	<0.001	17.1 ± 5.2	19.5 ± 4.8	<0.001
Perceived stigma	13.7 ± 3.1	11.0 ± 2.8	<0.001	13.4 ± 3.3	10.9 ± 2.7	<0.001
Substance abuse	101 (53.4%)	86 (45.6%)	0.04	109 (57.6%)	80 (42.4%)	0.01
Disease severity*	4.7 ± 1.0	2.5 ± 1.4	<0.001	4.2 ± 1.3	2.4 ± 1.5	<0.001
Medication side effects*	7.8 ± 2.1	3.6 ± 2.1	<0.001	7.1 ± 2.4	3.7 ± 2.3	<0.001
Chronic conditions	82 (37.4%)	137 (62.6%)	0.03	96 (44%)	123 (56%)	0.04

Table 4 shows the multivariable logistic regression analysis of factors associated with depression and anxiety. For depression, there were significant associations found for older age, female gender, divorced/widowed marital status, low income, perceived stigma, substance abuse, greater disease severity, more medication side effects, and the presence of chronic conditions. The odds of depression were two times higher for older patients compared to younger ones (adjusted odds ratio (AOR) 2.03, 95% confidence interval (CI) 1.01-4.05). Females had 2.5 times higher odds than males (AOR 2.5, 95% CI 1.1-6.0). Those divorced/widowed had nearly four times greater odds versus married patients (AOR 3.8, 95% CI 1.1-8.0). Low-income patients had 1.3 times higher odds versus high-income patients (AOR 1.3, 95% CI 0.9-1.9). Higher perceived stigma tripled the odds (AOR 3.2, 95% CI 1.1-5.3), while substance abuse doubled the odds (AOR 1.7, 95% CI 1.1-2.7). More severe MDR-TB disease and more medication side effects also significantly increased the odds of depression.

**Table 4:** Multivariate logistic regression analysis of factors associated with depression and anxiety * only for MDR-TB patients, p < 0.05: significant, AOR: adjusted odds ratio, CI: confidence interval, ref: reference category

Factor	Depression AOR (95% CI)	Anxiety AOR (95% CI)
Age	2.03 (1.01-4.05) *	1.02 (1.00-1.04)
Gender		
Male	1 (ref)	1 (ref)
Female	2.5 (1.1-6.0) *	1.2 (0.9-1.6)
Marital status		
Married	1 (ref)	1 (ref)
Single	1.1 (0.7-1.6)	1.0 (0.7-1.4)
Divorced/Widowed	3.8 (1.1-8.0) *	1.3 (0.8-2.1)
Income level		
Low	1.3 (0.9-1.9)	1.5 (1.0-2.2)
Middle	1.0 (0.7-1.4)	1.1 (0.8-1.5)
High	1 (ref)	1 (ref)
Perceived social support	0.9 (0.8-0.9) *	0.81 (0.71-0.86)
Perceived stigma	3.2 (1.1-5.3) **	2.2 (1.1-6.3) *
Substance abuse	1.7 (1.1-2.7) *	1.5 (0.9-2.3)
Disease severity*	1.8 (1.5-2.2) *	2.6 (1.3-4.0) *
Medication side effects*	2.4 (1.2-4.6) *	3.3 (1.1-5.5) *
Chronic conditions	1.3 (0.9-1.8)	1.1 (0.8-1.6)

For anxiety, significant factors included older age, low income, less social support, greater perceived stigma, more severe MDR-TB, and more medication side effects. Each year increase in age was associated with slightly higher odds of anxiety (AOR 1.02, 95% CI 1.00-1.04). Low-income patients had 1.5 times greater odds versus high-income patients (AOR 1.5, 95% CI 1.0-2.2). Lower social support and higher stigma also increased the odds substantially. MDR-TB severity and medication side effects again augmented the odds of anxiety.

Overall, the regression analysis identified vulnerable sociodemographic groups and modifiable psychosocial and clinical factors associated with poorer mental health in MDR-TB patients. These results can help guide targeted interventions to address high-risk patients' needs.

Table 5 depicts the qualitative themes, subthemes, and participant phrases (both MDR patients and HHC). This table presents qualitative themes, subthemes, and quotes on participants' experiences regarding mental health impacts, disease effects, stigma, psychosocial challenges, and suggestions. It provides complimentary personal perspectives to supplement the quantitative data.

**Table 5:** Qualitative themes, subthemes, and participant perspectives MDR-TB: multidrug-resistant tuberculosis, HHC: household contact

Theme	Subtheme	Participant quotes (MDR-TB)	MDR-TB phrases	Participant quotes (HHC)	HHC phrases
Mental health impacts	Depression symptoms	"I feel so hopeless about the future and can't stop crying." (Patient 103)	Hopelessness, crying spells	"I've noticed my loved one seems really down and cries a lot." (HHC 11)	Tearfulness, despondency
	Anxiety and worry	"My mind races with fearful thoughts about never getting better." (Patient 205)	Fear, rumination	"I've observed constant worrying in my family member." (HHC 02)	Nervousness, apprehension
	Emotional distress	"This illness has overwhelmed me emotionally." (Patient 306)	Emotional overload	"My family member feels emotionally drained due to this illness." (HHC 53)	Upset, overwhelmed
Stigma	Social isolation	"My friends have deserted me since I got MDR-TB." (Patient 124)	Social isolation	"Our social circle has distanced themselves since the diagnosis." (HHC 94)	Abandonment, loneliness
	Blame and judgment	"People act like I did something wrong to deserve this." (Patient 229)	Blame, judgment	"My family member faces judgment as if they caused this illness." (HHC 05)	Criticism, disapproval
Job/education issues	Education disruption	"I had to drop out of university because of this illness." (Patient 332)	Education disruption	"Our plans for education had to be halted due to the illness." (HHC 106)	School dropout, career interruption
	Loss of livelihood	"I lost my job and have no income now." (Patient 139)	Unemployment, loss of income	"Financially, things have become really tough for our family." (HHC 207)	Job loss, no earnings
Physical effects	Treatment side effects	"The drugs make me nauseous and cause severe headaches." (Patient 112)	Nausea, headache		
	Fatigue and weakness	"I'm constantly exhausted with little energy to even do daily tasks." (Patient 217)	Fatigue, weakness		
	Pain and disability	"The pain in my lungs makes it agony to breathe sometimes." (Patient 318)	Chest pain, breathing difficulty		
Financial hardships	Loss of livelihood	"I lost my job and have no income now." (Patient 139)	Unemployment, loss of income	"We're struggling to make ends meet after the main earner lost their job." (HHC 08)	Job loss, no earnings
	Treatment costs	"Paying for these medications has drained my family's savings." (Patient 243)	Financial burden	"The cost of medications is taking a toll on our family's finances." (HHC 309)	Monetary strain, depleted savings
	Increased expenses	"I'm struggling to keep up with health costs and medical diet." (Patient 347)	Increased expenses	"Managing the increased expenses has become a significant challenge." (HHC 10)	More costs, pricier diet
Family dynamics	Caretaking demands	"Caring for me is exhausting for my spouse." (Patient 157)	Caregiver burden	"I find it exhausting to care for my sick family member." (HHC 11)	Fatigue, burnout for spouse
	Risk of transmission	"I'm scared of passing the infection to my little kids." (Patient 262)	Transmission concerns	"I constantly worry about the infection spreading to my children." (HHC 121)	Fear of infecting children
	Worry for children	"I agonize over who will care for my children if I die." (Patient 375)	Childcare concerns	"Thinking about who will care for the kids if something happens is a constant worry." (HHC 13)	Concerns for child guardianship
Suggestions	Patient support	"Having a patient navigator to help with referrals would improve care." (Patient 127)	Patient navigation	"Support for family members in navigating the healthcare system is crucial." (HHC 148)	Care coordination assistance
	Integrated services	"Joint medical-counseling services would benefit treatment." (Patient 236)	Integrated care	"Combining medical and counseling services would be beneficial for families." (HHC 15)	Combined medical and therapy

## Discussion

The results of our study indicate a high and disproportionate prevalence of depression (37.5%) and anxiety (45%) among MDR-TB patients versus only 20% and 25%, respectively, in their household contacts. Of the diverse risk factors analyzed, perceived stigma, preexisting mental health disorders, and severity of disease and medication side effects emerged as consistent predictors associated with significantly greater odds of both depression and anxiety.

Our findings of elevated rates of mental health comorbidity in MDR-TB patients align with previous reports [12]. Globally, there is a wide variability in prevalence estimates, i.e., depression ranging from 45.6% to 71% and anxiety from 44% to 57%, across prior studies in Brazil, Indonesia, and China, echoing this inconsistency [13-15]. Our study is among the few to jointly assess dual outcomes of depression and anxiety and compare the burden between MDR-TB patients and their direct contacts.

The significant associations between mental health comorbidity and different sociodemographic, clinical, and psychosocial factors also corroborate many earlier findings, especially regarding perceived stigma, lack of social support, and substance abuse history [16,17]. However, our adjusted models highlighted disease-specific clinical parameters and preexisting psychopathology as stronger independent predictors. However, more reports have explored the relative impact of specialized correlates, such as MDR-TB treatment side effects, which are overlooked as contributors to mental health needs.

The themes of mental health impacts like depression, anxiety, and emotional distress echo results from prior research highlighting the psychological toll of MDR-TB. Participants described intense hopelessness, fear, and feeling emotionally overwhelmed. A regional ethnography also revealed profound grief and despair in the patient narratives. Feelings of isolation and despair appear to be common responses to an MDR-TB diagnosis [18,19].

In addition, the physical debility conveyed in the current study confirms previous findings. A scoping review found patients across drug-resistant TB studies reporting severe weakness, loss of appetite, nausea, and mobility issues from medications [20,21]. Our participants similarly highlighted agonizing treatment side effects and pain hindering daily functioning. The challenges of treatment regimens exacerbate patients’ physical health.

Finally, the suggestions for holistic patient support models align with global calls for integrating psychosocial services into MDR-TB care [22]. Participants recognized the need for mental health aid and decentralized community care to comprehensively address well-being. Qualitative insights substantiate the statistical data on mental health burdens and highlight priority areas for targeted interventions to alleviate patient suffering. Improving support across emotional, physical, and social realms will be essential to tackle the multidimensional impacts of MDR-TB.

Limitations and Recommendations

Several limitations should be considered when interpreting findings from this exploratory mixed methods study before making policy decisions. The consecutive sampling technique may limit the generalizability of results. In addition, the cross-sectional data cannot prove causal determinants of mental health outcomes. Self-reported measures are less objective than diagnostic interviews or medical evaluations. The lack of patient-contact linkage analysis misses household interconnections. Finally, health system challenges contributing to the MDR-TB burden were unaddressed. However, policymakers could still derive value in using actionable evidence on vulnerable groups requiring targeted mental health interventions. Going forward, research with randomized sampling, longitudinal follow-up, supplementary clinical data, household-pair analysis, and health systems perspectives would maximize robustness when shaping policies to build sustainable support systems for MDR-TB patients and families. Initial policy changes could develop integrated medical-counseling services prioritizing high-risk patients, while long-term policy reform might address modifiable structural factors, like poverty, stigma, or medication side effects exacerbating disease burden.

## Conclusions

The heavy burden of prevalent depression and anxiety among MDR-TB patients signals the imperative need for greater integration between NTPs and mental health services. Improved physical-emotional care coordination will be fundamental to patient-centric management and ending TB, especially among vulnerable groups. The intergenerational spillover to even household contacts underscores the importance of family-based approaches. In addition to clinical indicators, modifying perceptions of stigma should be a focus area for social interventions to alleviate dual disease impacts.
